# Fondazione Telethon and Unione Italiana Lotta alla Distrofia Muscolare, a successful partnership for neuromuscular healthcare research of value for patients

**DOI:** 10.1186/s13023-021-02047-1

**Published:** 2021-10-02

**Authors:** Anna Ambrosini, Danila Baldessari, Silvia Pozzi, Manuela Battaglia, Elena Beltrami, Anna Maria Merico, Marco Rasconi, Lucia Monaco

**Affiliations:** 1 Fondazione Telethon, Via Poerio 14, Milan, Italy; 2 B.E.A. Consulting, Milan, Italy; 3 UILDM, Unione Italiana Lotta alla Distrofia Muscolare, Padua, Italy

**Keywords:** Neuromuscular disorders, Healthcare research, Patient empowerment, Trial readiness, Innovative therapies

## Abstract

In 2001, Fondazione Telethon and the Italian muscular dystrophy patient organisation Unione Italiana Lotta alla Distrofia Muscolare joined their efforts to design and launch a call for grant applications specifically dedicated to clinical projects in the field of neuromuscular disorders. This strategic initiative, run regularly over the years and still ongoing, aims at supporting research with impact on the daily life of people with a neuromuscular condition and is centred on macro-priorities identified by the patient organisation. It is investigator-driven, and all proposals are peer-reviewed for quality and feasibility. Over the years, this funding program contributed to strengthening the activities of the Italian neuromuscular clinical network, reaching many achievements in healthcare research. Moreover, it has been an enabling factor for innovative therapy experimentation at international level and prepared the clinical ground to make therapies available to Italian patients. The ultimate scope of healthcare research is to ameliorate the delivery of care. In this paper, the achievements of the funded studies are analysed also from this viewpoint, to ascertain to which extent they have fulfilled the original goals established by the patient organisation. The evidence presented indicates that this has been a highly fruitful program. Factors that contributed to its success, lessons learned, challenges, and issues that remain to be addressed are discussed to provide practical examples of an experience that could inspire also other organizations active in the field of rare disease research.

## Background

Fondazione Telethon (in brief: Telethon) is a non-profit organisation recognised by the Italian Ministry of Education, University and Research, that finances research on genetic diseases, focusing on those that for their rarity are not a priority in health policy and drug development [1]. It was founded in 1990 out of the will of the Italian muscular dystrophy patient organisation (PO) Unione Italiana Lotta alla Distrofia Muscolare (UILDM), which urged to start research towards the cure of genetic muscular dystrophies and myopathies. The immediate success in fundraising prompted the Telethon Board of Directors in 1992 to extend its mission to all genetic diseases. Telethon’s funding decisions are based on two principles: research must be scientifically excellent and must address the patients’ mandate to develop a therapy for their disease and make it available to them. The fulfillment of this vision for the therapeutic approaches in the Telethon’s pipeline that reached the clinical stage implied the development of strong business development and regulatory affairs competences. In 2010, Telethon established the first relevant partnership with industry (GlaxoSmithKline, “GSK”), leveraging the results of its investment in gene therapy started in 1995 with the creation of the San Raffaele-Telethon Institute for Gene Therapy in alliance with the San Raffaele Hospital in Milan. Such a multi-stakeholder collaborative approach was instrumental in completing the path starting from basic research to clinical studies and led in 2016 to the marketing authorization by the EMA for Strimvelis, a gene therapy for the treatment of adenosine deaminase deficiency immunodeficiency a rare monogenic disease. This was the first ex vivo gene therapy treatment ever approved [1]. At the end of 2020, Libmeldy, another gene therapy medicine derived from the Telethon’s pipeline, was approved by the European Medicine Agency for treatment of children with metachromatic leukodystrophy [2]. Four other gene therapy treatments are in the Telethon clinical pipeline (not shown). Overall, 130 patients from 32 different countries have been treated with these gene therapy approaches.

Despite the expansion of its mission to all rare genetic diseases and the development of successful gene therapy programs, neuromuscular disorders (NMDs) have always remained at the heart of the Foundation’s objectives, with a total investment of about 125 million Euros in this research area (about 23% of total investment).

NMDs are a highly heterogeneous group of diseases, in terms of epidemiology, genetics, molecular physiopathological mechanisms, and clinical phenotypes [3, 4]. When, in the late nineties, the gene therapy programs at Telethon entered a new age for those diseases for which hematopoietic stem cell correction was feasible [1], it became clear that this therapeutic strategy would not be easily translatable to NMDs. First of all, such gene therapy protocol was not suitable for muscular dystrophies, where the genetic correction must be delivered directly to the muscle tissue. Moreover, in the late nineties the understanding of the mechanisms of muscle degeneration/regeneration mechanisms was still quite limited, as was the clinical knowledge of the different NMDs. At that time, Telethon-supported NMD research was primarily seeking to fill this gap through basic studies focused on gene discovery and mechanistic understanding of the muscle physiopathology. Clinical study designs suffered from important limitations, mainly due to a lack of knowledge of natural history and availability of appropriate functional measures, as well as to insufficient statistical power and poor networking to overcome these problems. Overall, this research was very far from having a significant impact on people affected by muscular dystrophy. Clearly, the NMD field required a research strategy different from the path developed for the Telethon gene therapy programs and UILDM strongly called for a new approach to address patient needs.

To address the above limitations, Telethon and UILDM together designed a call for clinical research grants exclusively dedicated to research aimed at improving the quality of life (QoL) of people living with a neuromuscular condition. In 2001, Telethon launched the first call of this program and has since issued it regularly, with UILDM providing annual financial support of around 600,000 euros. This program has also attracted additional funds from other patient organisations and pharmaceutical companies interested in the NMD field. At the end of 2020, the total investment in this strategic program was 12.37 million euros.

Over the years, this initiative has been instrumental to translate clinical observations into healthcare practice and implement a holistic approach to the person’s medical needs. Furthermore, the results of the Telethon-UILDM clinical grant investment have been enabling factors for the translation of innovative therapeutic approaches developed by international pharmaceutical companies into clinical research and therapies, now available to patients.

This paper focuses on the research management model adopted by Telethon for this special program, from the rigorous peer review to select excellent clinical projects to the lessons learned in managing the grants, particularly those dedicated to multicentre projects. The impact of the increased clinical knowledge on patient care and on the development of the NEuroMuscular Omni (NEMO) centres, multispecialty clinical centres fully dedicated to patients with NMDs [5, 6], is discussed, as well as the facilitating role of this gained knowledge for the implementation of innovative therapies. The key factors that contributed to the success of this program and issues that still require consideration are analysed. This experience is shared in the hope that it will also inspire other charities and POs interested in funding clinical research on NMDs or, more generally, on rare diseases.

## Methodology

### Project selection and management

Applications submitted to the Telethon-UILDM Calls for NMD clinical projects underwent a rigorous peer-review evaluation, involving international clinical experts. Advice from consultants with expertise in biostatistics was made available to interested investigators to improve the study design during the preparation of the applications. Occasionally, additional grants were awarded to the NMD clinical network with financial support from other NMD POs or pharmaceutical companies; these ad hoc sponsored projects also underwent a rigorous peer review to assess their scientific quality and feasibility before awarding grants. Periodic analyses were conducted by the Telethon scientific office to identify critical issues in the management of the funded projects and implement corrective actions to counter failures or prevent those threats that mainly affect multicentre studies’ performance. The focus of the call was also periodically revisited together with UILDM.

### Bibliometric analysis

A total of 354 publications (original articles, letters and brief case reports, and reviews) acknowledging the Telethon-UILDM studies and/or the other related ad hoc clinical grants was indexed through 2021–01-31 (source: Telethon publications’ database, powered via the Web of Science Core Collection™ onto the Web of Science platform by Clarivate™ [7] and via the Europe PubMed Central platform [8]; last access to both 2021–02-26). The original articles and the reviews (348 publications) underwent a bibliometric analysis based on the relative citation ratio (RCR), a metric developed by the National Institutes of Health (NIH) Office of Portfolio Analysis [9]. RCR is an article-level metric calculated as the number of cites per year of each paper, normalized to the citations per year received by NIH-funded papers in the same field. Fields are sampled for each article by using its co-citation network. The RCR values are provided through the *iCite* web interface made available by NIH [10]. The RCR value of 320 of the 348 publications was available on the *NIH* platform, being the other 28 papers too recent or lacking the PubMed Identifier required for the analysis (values calculated on 2021–03-19).

Some key publications acknowledging the funded studies are presented in more detail in the Results and Discussion sections. The selection was based on one or more of the following criteria: i) relevance for the topic discussed; ii) tangible evidence of healthcare implementation; iii) pivotal paper or most recent publication of the author on the topic; iv) RCR value above 1 (if available).

### Classification of the medical fields and topics

The assignment of the main field category and specific topics of each project is based on the keywords indicated by the principal investigators in their original application, chosen from among a set of medical fields and research topics provided within the Application form, and subsequently validated by the Telethon scientific office.

### Customer satisfaction of patients admitted to the NEMO centres

Aggregated data from Customer Satisfaction questionnaires of the NEMO centres were available online within their most recent annual Impact Report referring to the year 2019 (pages 41 to 45) [6]. In that Report, items based on a standard scale were grouped according to specific areas of the patient’s perceived quality of care and average scores were given based on a ranking that was calculated by the NEMO professionals over a range of 1–10 [6]. These results are herein briefly reported together with the NEMO vision illustrated at page 12 of the same Impact Report (after translation in English and back translation for content validation) [6].

## Results

### A call for grant applications to prioritise NMD healthcare research

The Telethon-UILDM Call initiative has been dedicated to NMD healthcare research aimed at improving patient daily life. UILDM representatives identified the main topics of interest to them and defined the research priorities, which were periodically revised, while the Telethon research managers implemented rigorous methods for the selection and monitoring of the projects. The strategic definition of the program also benefited from the advice of international experts in the NMD clinical field. The call for projects aimed at developing diagnostic, preventive, therapeutic and rehabilitative approaches for NMD of genetic origin in the following main medical areas of interest: cardiology, internal medicine, neurology, orthopaedics, psychology and respiratory medicine. Other topics that UILDM considered to be of great relevance concerned assistance outside the hospital context, and support and training to caregivers. Collaboration between different clinical centres and participation in multicentre studies have always been strongly encouraged. The quality of grant applications, as measured by the calls’ success rate, has significantly improved over the years, also thanks to the methodological support offered by Telethon and the careful evaluation and the constructive suggestions provided by the reviewers to the applicants. In the period 2001–2019, 14 calls were issued, with a total of 56 grants awarded. The average number of applications received per call was 13 (range 9–20; median 14), with 4 projects (range 2–6; median 4) approved for funding (mean success rate of 30%). Five additional ad hoc grants were also awarded to consolidated projects after a rigorous peer review evaluation, to provide continuity to the ongoing clinical research. These 5 granted projects have also been included in the analysis herein presented. The total investment in this program has been 12.37 million euros.

**Fig. 1:**
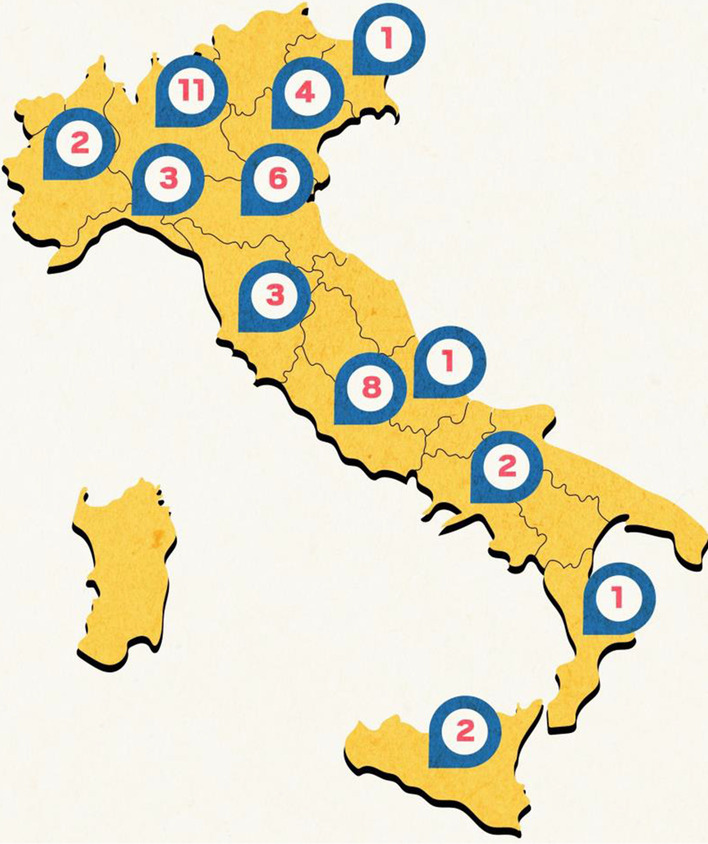
Distribution of the Italian NMD clinical centres awarded with Telethon-UILDM grants. In circles: number of centres per region

Among the 61 granted projects, 46 were collaborative studies, with a mean number of 8 clinical centres per multicentre project (number of centres in the range 2–15; median 8). Overall, 44 tertiary clinical centres in Italy (Fig. 1) and 120 principal investigators actively participated in these projects.

As expected, neurologists represented the vast majority of specialists involved. Nevertheless, other experts participated in many projects, both as coordinators (cardiologists, endocrinologists, medical geneticists, nutritionists, psychiatrists, psychologists, or bioengineers), and as partners (pulmonologists, radiologists, experts in sleep medicine, or biostatisticians).

After grant approval, Telethon carefully managed funds and monitored the performance of the projects, with special attention to the multicentre efforts. Continuous dialogue with the project coordinator and periodic analyses allowed to identify several critical issues, which were mainly related to: (i) extension of trial duration; (ii) lack of standardisation of operations and differences in the adoption of standards of care among centres; (iii) centre over-commitment across multiple projects (Table 1). Corrective actions were put in place by Telethon to counteract failures or prevent those threats that mainly affect multicentre studies, adopting more stringent eligibility criteria within the calls and better-defined administrative rules, and working closer to the coordinators to support their project management (see a few examples in Table 1). This process greatly contributed to harmonising the networking activities and fostered a cultural shift towards best practices in clinical research and data sharing.

**Table 1:** Telethon management of criticalities in the conduct of multicentre clinical trials

Critical issues (causes and consequences)	Actions taken
*Extension of trial duration*	*Administrative management of the grant*
* Time lag among centres for Ethics Board approval	Start of the study only when all centres are ready
* Difficulties in patients’ enrolment	Administrative distinction between start-up/follow up (fixed) costs and “per patient” (variable) costs, with funds on variable costs allocated only to performing centres, based on periodic reports on patient enrolment and follow-up
** Expanded recruitment time and length of the study	
** Insufficient statistical power; inclusion of additional centres; protocol amendments; lack of funds	
*Lack of standardisation of operations*	*Management support to Coordinator*
* Uneven execution of functional measurements between centres	Clinical monitor support and good clinical practice compliance assessment
* Unequal data quality and poor case report form maintenance	Request for stronger coordinator management and training on outcome measures and data collection
* Poor awareness of data protection principles	Regular periodic meetings with the study steering committee
** Clinical data provided by the centres not comparable	Regular periodic reports to Telethon
** Lack of secure centralised systems for data management	Centralised IT platform available for patient registries and standard operating procedures for data sharing
*Centres’ over-commitment*	*Rules of the grant applications*
* Lack of dedicated personnel	Limitation in the number of active studies in which an investigator can participate
* Overlap with routine clinical activities	Cross-check of the number of staff full time equivalents reported in the applications
** Inadequate number of professionals to ensure patient follow-up on schedule	

In the column “Critical issues”: * Indicates “cause”, ** Indicates “consequence”

### Diseases and medical fields addressed by the funded projects

**Fig. 2:**
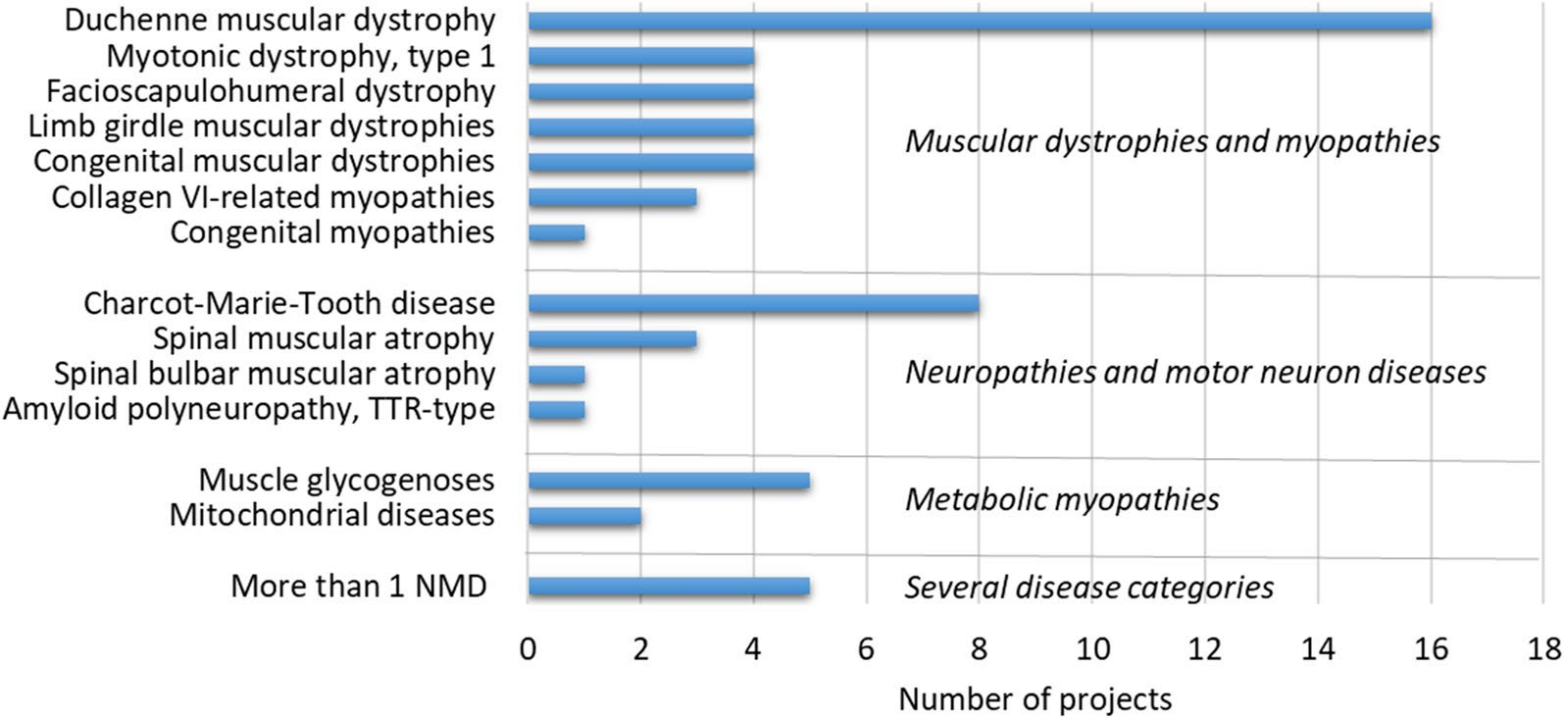
Diseases addressed by the funded projects. The graph indicates the number of projects addressing a specific disease/disease group or multiple diseases. Diseases are organised into 4 main broad categories, namely: (i) muscular dystrophies and myopathies; (ii) neuropathies and motor neuron diseases; (iii) metabolic myopathies; (iv) several diseases. *NMD* neuromuscular disorder; *TTR* transthyretin

In general, each study addressed a single disease/disease group, with dedicated clinical working groups, who have also been successful in obtaining follow up grants through this funding program and have progressed on trial readiness in their specific field. Duchenne muscular dystrophy (DMD) and Charcot-Marie-Tooth disease (CMT) were the most studied diseases, followed by muscle glycogenoses and subgroups of muscular dystrophies other than DMD and spinal muscular atrophy (SMA) (Fig. 2).

Only 5 studies focused on more than one disease group, addressing the development and the validation of functional outcome measures (OM) or QoL scales across different conditions (Fig. 2).

Overall, more than 6000 patients were involved in these studies. At the time of submission of this manuscript 5 studies, all multicentre ones, were ongoing.

The funded projects addressed different medical fields, namely: cardiology, endocrinology and nutrition, genetics, neurology, physical medicine and rehabilitation, and psychology and QoL. Table 2 reports the main topics addressed within each area, the number of funded projects and the number of derived peer-reviewed publications.

**Table 2:** Medical fields and main topics addressed by the funded projects

Medical field	Main topics	No. of projects	No. publications; Period: 2004–2020 (Sources: Web of Science platforms by Clarivate™ and Europe PubMed Central)
Cardiology	Diagnosis; prevention; device; precision medicine; biobanks	4	13
Endocrinology and nutrition	Bone density; body mass index; body composition; dietary	3	7
Medical genetics	Genetic diagnosis; gene panels	7	53
Neurology	Geno-phenotype correlation; functional outcome measures; natural history; standards of care; pharmacological trials	38	240
Physical medicine & rehabilitation	Exercise training; assistive technology	4	21
Psychology and quality of life	Quality of life questionnaires; caregiver burden; neuropsychological and psychiatric evaluation	5	20

### Bibliometric analysis

Overall, the projects generated a comprehensive body of information, testified by 354 peer-reviewed publications (Web of Science platform by Clarivate™ and Europe PubMed Central [7, 8], including original articles (n = 316), letters and brief case reports (n = 6), and reviews (n = 32) (Table 2).

Bibliometric analysis was performed on original papers and reviews only. Telethon signed and endorses the San Francisco Declaration on Research Assessment (DORA) [11] and does not apply journal-based metrics even to assess candidates applying to its call for grant applications. Instead, the RCR metric of the NIH Portfolio office [9] was adopted as indicator of scientific influence in our bibliometric analysis. This metric is article-level and field-independent and identifies publications that are influential in the peer co-citation network, against a benchmark of papers derived from NIH projects, where RCR ≥ 1.0 and ≥ 2.0 correspond to the NIH top 50% and 25% respectively. The RCR values of 320 publications (original articles and reviews) were available on the NIH *iCite* web platform [10]. RCR mean was 1.69 +/− 0.12 and median 1.1, with 180 publications having RCR > 1.0, including 81 with RCR > 2.0. Overall, these numbers suggest that, as a whole, the publications derived from Telethon-UILDM and the other related ad hoc clinical projects performed similarly or slightly better than the NIH benchmark in terms of influence on their peer community, with 56% and 25% of them falling in the NIH top 50% and 25% respectively. A striking difference was noted between original articles (RCR mean 1.47 +/− 0.08 and median 1.06; n = 288) and reviews (RCR mean 3.63 +/− 0.89 and median = 1.62; n = 32). We also evaluated if RCR of publications in the different medical fields were comparable. Although for some of them the total number of projects—and acknowledging papers—was low, it is interesting to note that the RCR mean and median were above 1 for all groups but one, suggesting comparable publication recognition across the medical fields (Table 3), with a notable 1.88 mean RCR value for the neurology field, gathering two thirds of the publications analysed.

**Table 3:** RCR values of publications grouped by medical field of the projects

Medical fields	RCR mean +/− sem	RCR median	No. indexed publications; Period: 2003–2020 (source: iCite)
Cardiology	0.93 +/− 0.15	0.91	9
Endocrinology and nutrition	2.09 +/− 0.60	1.22	5
Medical genetics	1.27 +/− 0.16	1.04	50
Neurology	1.88 +/− 0.17	1.15	220
Physical medicine and rehabilitation	1.17 +/− 0.13	1.01	20
Psychology and quality of life	1.07 +/− 0.13	1.1	16

*RCR* relative citation ratio

The relatively higher number of publications from projects in this field (220) allowed additional analysis, which was separately conducted on original articles and reviews. The percentage of papers with RCR ≥ 1.0 was 56% (n = 125 of 220), in line with the whole publications’ analysis. Interestingly, the RCR of the reviews was 4.31, a value which falls in the NIH top 10% (Table 4). Among them, 7 reviews scored above 4 (RCR range 5.16–26.02; median 8.66), with 6 having the Telethon principal investigator as first or last author (not shown). Overall, 57 publications in the “neurology” field derived from international collaborations, 41 of which with the Italian principal investigator as first or last author (Table 4), attesting the key role of the Telethon-funded investigators also in the international context.

**Table 4:** RCR values of original articles and reviews in the field of neurology

	Original articles (n = 197)	Reviews (n = 23)
Mean RCR +/− sem	1.61 +/− 0.12	4.31 +/− 1.19
Publications with RCR ≥ 1.0 (NIH top 50%)	107	18
Publications with RCR ≥ 4.0 (NIH top 10%)	14	7
International collaborative papers	48	9
Italian authorship in International collaborative papers	36	5

*NIH* National Institutes of Health (USA), *RCR* relative citation ratio

### Outcomes of the studies

Projects dedicated to genetic diagnosis and geno-phenotype correlation led to the characterization of large disease-specific patient cohorts [12–20]. In addition to increasing disease knowledge, these studies contributed to identifying patients and families still missing a genetic diagnosis that underwent new genetic examination, with many families receiving appropriate genetic counselling [21–24]. Some studies allowed the identification of life-threatening risks, such as the mutations in the transthyretin (TTR) gene, which cause familial amyloidosis with severe cardiological and neurological dysfunctions [25]. The genetic testing of proband’s relatives and the adoption of good practices to monitor even subtle clinical changes in asymptomatic carriers are particularly relevant for familial amyloidosis of TTR-type given the recent availability of effective innovative treatments [26].

The functional studies contributed to the timely update of OM under the umbrella of international consortia such as the Translational Research in Europe—Assessment and Treatment of NeuroMuscular Diseases (TREAT-NMD) network [27–29] and the Inherited Neuropathy Consortium [30–32], or promoted the development of new patient-centric OM with the direct involvement of patients and POs in their definition [33–35]. In addition to being adopted as functional endpoints in international trials [36, 37], these measures have been used to monitor current treatments [38] and were introduced into clinical routine practice, becoming a relevant tool as part of the standards of care [39].

Other studies addressed specific daily life needs, exploring QoL and caregiver burden of people with NMDs [40, 41] or gathering information on nutrition and body composition, neuropsychological evaluation, physical exercise, pulmonary function, sleep disturbance, etc. (Table 1). In general, these were multicentre projects and involved specialists other than neurologists. Innovative studies based on brain-computer interface, gate analysis or exoskeletal devices were designed and implemented by neurologists and biomedical engineers working in close collaboration with patient groups. This approach was established upfront and was included in the design of the proposed applications.

A few clinical trials testing the efficacy of small molecules were also supported through this funding program. These concerned: a double-blind randomized, placebo-controlled, pilot trial of Ramipril in McArdle's disease [42]; a double-blind randomised ascorbic acid in CMT type 1A [43]; an open study on combined enzyme enhancement therapy and enzyme replacement therapy in Pompe disease [44]; a pilot open study in Collagen VI-related myopathies with Cyclosporine A [45].

### Registries and real-world data from approved treatments

Clinical data contributing to define the disease natural history have been collected within highly structured patient registries [46], based on an information technology platform deployed according to a privacy by design model compliant with the current European General Data Protection Regulation 2016/679 [47]. The data stewardship is held by a legal entity including Telethon, UILDM and other Italian NMD POs and is based on transparent governance and data use agreements with the involved clinical centres [48]. Distinct datasets collect genetic and clinical information on SMA, CMT, familial amyloidosis of TTR-type, muscle glycogenoses, spinal and bulbar muscular atrophy, congenital myopathies, and muscular dystrophies (congenital, limb girdle, and facio-scapulo-humeral dystrophy types). These registries include both clinician-reported [25, 48] and patient-reported [49, 50] forms. Aggregated data derived from the NMD registry were also provided to industry for feasibility studies, establishing a transparent process of data sharing that contributes to the registry sustainability (unpublished). Moreover, in 2018 this platform has been made available to clinicians for post-marketing SMA data collection. Another registry on mitochondrial disorders was started with a Telethon-UILDM grant and is managed directly by Mitocon, the Italian mitochondrial PO, on a different information technology platform [51]; its data already contributed to several studies and publications (see, for example, refs [15] and [52]).

### Natural history data to support industry trial design and interpretation of study results

Thanks to the support of several consecutive Telethon-UILDM grants, the Italian clinical network collected longitudinal clinical and functional data from a large cohort of children with DMD, which helped define the natural history of this disease. These data contributed to steering the international discussion on DMD therapy approval with all DMD stakeholders and with regulators [36, 53]. The availability of this accurate data collection attracted the interest of pharmaceutical companies engaged in the development of new therapeutic approaches for DMD. The experience gained by Telethon in its previous relationship with industrial partners was instrumental in establishing data use agreements for nonexclusive use of anonymised patient data by companies (not shown) or by the “collaborative Trajectory Analysis Project” (cTAP), a pre-competitive coalition of different DMD stakeholders aimed at understanding the high variability in clinical trial outcomes [54]. Notably, the trajectories of ambulatory function measured by the 6-min walking distance test have been used in an international context to define the best statistical model to represent natural history and explain the variability in disease progression [54]. The resulting patients’ subgrouping, which considers age, steroid use and 6-min walking distance baseline values, can help explain variations in trial results and support future trial design [54]. Functional trajectories derived from the Italian DMD natural history studies also helped establish that disease progression differs in patients with different deletions amenable to different exon skipping [55]. Moreover, they have been recently used for meta-analyses also including cohorts of DMD patients from other countries, demonstrating that data derived from real-world and natural history are comparable with those collected in the placebo arms of 6 different trials [56]. Based on this evidence, the authors further suggested that natural history/real world data of patients could be considered as external controls in trials, thus avoiding the use of a placebo group, when patients are matched for genetic and clinical characteristics, and the same standards of care are adopted [56].

### Implementation of standards of care

The synergistic activity among the Italian NMD clinical centres promoted by the Telethon-UILDM grants and the concomitant participation of this clinical network in international research efforts contributed to disseminating among the tertiary clinical centres the most updated guidelines on standards of care, where available [57–59], highlighting the need of implementing a multidisciplinary, patient-centric, approach to patient care.

In line with this holistic approach, in 2007 UILDM and Telethon promoted a new clinical centre model, fully dedicated to patients with NMDs, the NEMO centre, which is now operating through 6 centres located in different parts of Italy [5, 6]. These clinical centres are based on a public–private model and on a governance that includes NMD POs to guarantees greater flexibility in the priority setting and management. The vision of the NEMO centres is “based on the need for a global and continuous care of the patient, both in adult and pediatric age, with a view to achieving the best quality of possible life, through personalized rehabilitation programs. Everything revolves around the person and his family. That's why the NeMO center is not only a place of care, but also a home where people of all ages and their families are welcomed, to listen, support and accompany them throughout their life path” [6]. The NEMO centres collect the voice of patients and their perceived impact of care through Customer Satisfaction questionnaires, the most recent data of which are reported in the NEMO annual Impact Report 2019 in aggregated form [6]. A very high score (9 or higher in a scale 1–10) was assigned by patients to all groups of items, namely: (a) patient-centricity; (b) trust in medical competence; (c) two-way dialogue; (d) continuity and home care support; (e) time in waiting list [6]. The NEMO centres have also participated in many of the clinical research projects herein described, either as coordinators or partners.

Several centres of the Italian NMD clinical network participate in the European Reference Network for Rare Neuromuscular Disease (Euro-NMD ERN), aimed at “harmonizing and implementing standards for clinical and diagnostic best practice, improving equity of care provision” across Europe [60].

## Discussion

The value of an investment in biomedical research should be estimated by measuring the level of excellence and innovation of its outcomes, ultimately leading to effective medications accessible to patients [61]. This is also the vision of Telethon, which for the past 20 years has worked to make gene therapy treatments in its pipeline accessible to patients [1, 2]. Fulfilling the mandate of patients affected by NMDs, however, has implied developing a different program of investments, aimed primarily at addressing the daily life demands of people with a neuromuscular condition. While the scientific quality of the clinical research proposed by the investigators was a key requirement for the funding decision, it was more difficult to identify the right indicators to measure the excellence of outcomes of this research-based health program that addressed medical needs of everyday life. On the one hand, health research may not imply innovative science, while retaining its important value for patients. On the other hand, there is a research-to-practice gap between the evidence produced by healthcare research and its effective translation into practice to improve patient care and treatments [62, 63]. How, then, to evaluate the effectiveness of a strategic program such as the one herein described in terms of excellence and ability to achieve its original objectives? The research outcomes have been analysed from two different angles, the impact on clinical knowledge and the impact on patients’ daily life, two sides of the same coin, as their value to patients should ultimately converge.

### Harmonisation of clinical research activities

Through the years, the community of the Italian clinicians has responded with great interest to the Telethon and UILDM joint call to action and, undoubtedly, the program produced significant outcomes and cultural changes. Thanks to this initiative, a strong clinical network, which includes most of Italian NMD tertiary clinical centres, has been consolidated (Fig. 1). Clinical scientists have strengthened their research on different NMDs (Fig. 2) and contributed to NMD trial readiness in a timely fashion against the international scenario or even anticipating research needs, for instance regarding development and validation of OM [28, 29, 31–35]. They engaged in longitudinal data collection to increase knowledge on natural history for several NMDs, and standardised methods to evaluate patients [36–39]. They identified and challenged new pharmacological [42–45] or physical [31] therapy approaches and collected information on psychosocial burden to implement support actions [41, 50]. By presenting the ongoing results during the scientific meetings and POs’ congresses, they promoted the sharing of information and new ideas. Moreover, through dissemination of the outcomes of these studies, they have contributed to building international consensus on clinical trial design and standards of care guidelines. A large number of peer-reviewed publications was produced, with relatively high RCR scores, which are indicators of a strong influence on the NMD peer community (Tables 1, 2, 3, 4).

### The value for patients

**Fig. 3:**
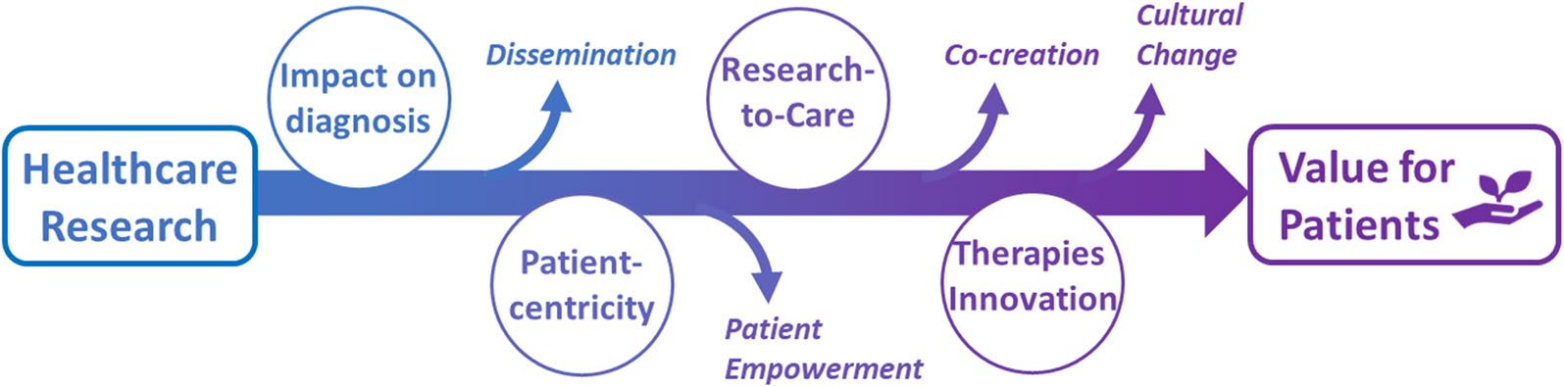
Domains that concur to evaluate the value of the investment in healthcare research. In circles: domains with direct impact; indicated by the small arrows: domains of indirect impact

Looking at numbers and publications is not enough to establish the real value of healthcare research, whose ultimate scope is to be translated into practice and improve patient QoL [62, 63]. This investment was therefore evaluated for its impact on patients, by analysing to which extent the outcomes of the granted studies have been translated into medical practice or have promoted the development of innovative therapies. Figure 3 summarizes the domains that may contribute to creating value for patients, either directly (circles) or through an indirect, albeit relevant, manner (small arrows). For each domain strengths and open issues are highlighted below.

#### Impact on diagnosis

The genetic and clinical characterisation of large patient cohorts has had a broad impact nationwide on providing genetic diagnosis, inform about prognosis, and facilitate genetic counselling and decision making [12–25]. The investment in genomic research allowed to identify new mutations and new genes not yet included in the genetic services provided by the national health system, exploring what Horton and Lucassen defined “a hybrid space where clinical practice and research need to co-exist” [64]. Progress in molecular diagnosis has also benefited from nationwide sharing of information and patients’ samples carried out by centres as part of the projects’ activity. Despite these achievements, however, many patients/families still wait for the molecular characterization of their disease [65, 66]. Patients with late-onset/slow-progression forms may also miss a competent tertiary centre, thus experiencing diagnostic delay even in case of well-characterized diseases such as SMA [67]. Furthermore, available information on diagnosis and prognosis is essential but not sufficient for real shared decision-making, if effective communication is not established between healthcare professionals and their patients; much remains to be done both by professionals and POs to promote together this cultural change [62, 68].

#### Patient-centricity

With this particular investment, Telethon and UILDM meant to stimulate clinical researchers to approach medical research in a more person-centred manner. Some studies directly involved patients and caregivers, for instance, engaging them in surveys on QoL, disease burden and needs for psychosocial support [40, 41, 50], and collection of information on their medical requirements [15, 49, 52]. POs focused on DMD and their patient representatives participated in the development of a new functional scale that reflects patient mobility needs and daily life activities [33, 34] and patient-reported functional OM [35]. These are examples of a general trend of direct consultation with POs and patients during study design and delivery that has become more frequent, although it is not the norm yet [68].

#### Transforming research into care

To carry out careful natural history studies, more sensitive functional measures to monitor disease progression had to be developed and up to date standards of care implemented to standardise patient evaluation among centres. Consequently, best care practices have been disseminated nationwide and the centres participating in the studies became familiar with them, supported also by disease-specific clinical care guidelines derived from international consensus [57–59].

Although also these guidelines emphasise the need for multidisciplinary care in the management of patient with NMDs, the degree of its actual implementation still varies from centre to centre. One reason is that putting it into practice is often beyond the doctor's willingness, as this process can also depend on additional factors, such as organizational norms, local and regional health policies, and policymaker decisions [63]. For these reasons, UILDM and Telethon in 2007 founded the NEMO clinics fully dedicated to patients with NMDs, which now count 6 active centres in Italy [5, 6]. The NEMO centres are built on the concept of multidisciplinary care and person-centric holistic approach and have a high index of appreciation by patients for the care provided [6]. Although they work towards full integration of clinical research and medical care [69], their main challenges are related to the limited personnel to participate in numerous international trials, while also handling high medical demands. Multidisciplinary assistance has also been implemented by Italian NMD clinical centres beyond NEMO, with the main difficulty being the need to logistically fit this care approach within the general hospital context.

The demonstrated capability to provide quality assistance made many of these centres eligible to be included in the Euro-NMD ERN [60].

#### Innovation and therapies

The increased clinical knowledge in the field of DMD and SMA gained thanks to the Telethon-UILDM projects contributed to the clinical trial design for innovative therapies, working on OM adopted in international trials [36, 37] or contributing to a new statistical approach for trial data evaluation [54–56]. Telethon made agreements to share data for nonexclusive use with third parties, including industry. Other agreements with industry were based on unconditional support provided to Telethon to make the long-term natural history data collection sustainable. Natural history data collected by the Italian DMD clinical network supported international efforts focused on the interpretation of clinical trial data [36, 54–56] and contributed to the discussion with regulators [53].

Moreover, the high level of standardisation of the clinical measures and care reached by several Italian NMD centres has been an enabling factor to favour their inclusion in international trials testing innovative therapies on DMD and SMA, allowing the participation of many Italian patients in these trials. Several centres, including NEMO centres, also participated in early access to therapy programs, i.e. for SMA [70] and are now collecting real world data into highly structured registries.

#### Cultural change

This path towards “Value to patients” has had an indirect impact on other domains as well (Fig. 2), such as dissemination of good practices on data sharing, better communication between clinicians and POs, and patient empowerment, all ultimately contributing to a cultural shift towards the involvement of multiple stakeholders in a co-creation process on healthcare research. Notably, not only UILDM has been the driver for the ideation, development, and funding of this clinical research program, but it also became a main actor in the development of the unique models of the NEMO clinical centres [5, 6] and of the legal entity that holds the stewardship of the NMD registry platform [48]. The example of UILDM has been followed by other POs that contributed to support the clinical research programs dedicated to their disease of interest, and/ or are partners in the NEMO enterprise and the NMD registry platform initiative.

### Key factors for the success of the initiative

A rigorous peer review process allowed to support the research activity of many excellent clinical scientists, who enthusiastically participated in the funded projects bringing new ideas and their leadership and expertise. Continuity of the financial support and strong management implemented also considering the lessons learned contributed to the growth of a strong clinical network. Although public resources have always been rather limited, the Italian national health system has provided support, with structural funds allocated to the clinical units, for instance for the salary of principal investigators and to cover biomedical examinations that are part of the routine patient care. The support provided by NMD POs, not only by providing funds and structural platforms, but also through continuous dialogue and sharing of knowledge was fundamental for the implementation of the studies.

In the last decade, the international scenario on clinical research on NMDs has changed radically, with a big step forward in trial readiness and with the advent of innovative therapeutic approaches for diseases such as DMD and SMA [4]. This focused funding program provided the opportunity for Italian experts to contribute to an international cross-fertilisation process by leveraging a vast clinical network and patient cohorts nationwide and for Italian patients to participate in international trials and benefit from early access to therapies.

### Challenges and opportunities

Although the Telethon-UILDM initiative was based on identified priorities and focused on specific diseases and topics, essentially it has been an investigator-driven extramural research funding. A typical consequence of bottom-up investments is that funders’ priorities do not always match those of investigators, who propose research that better suits their expertise and interests [71]. This was at least partially the case with this program, where some important issues of great relevance to the NMD patient community remained unaddressed, such as implementation of home care support or management of specific medical needs. One way out is to set strict priorities in advance by clearly explaining in the call for grant applications what kind of research funders are willing to support, although this does not guarantee a positive and successful response from applicants. In the attempt to steer the research agenda and attract new medical competences within the clinical network, the call has been revised over the years, becoming more focused on the diseases and medical fields most relevant to UILDM.

Running clinical studies that involve large research networks remains a challenge due to the limitation of economic resources that can be invested annually. A sustainability plan that has already proven its feasibility has involved partnering with pharmaceutical companies interested in developing therapies for NMD, particularly regarding the collection of data that may be relevant to regulators’ decision making [53–56]. This multi-stakeholder approach would be a convenient opportunity for all parties, but it also implies a cultural shift towards unconditional support by companies to academic clinical activities that are essential to prepare the ground for future trials, where return may not be immediate [72]. Importantly, it can also contribute to promoting training and career development of junior clinical scientists, preparing the ground for the clinical research of the future.

### Limitations of this analysis

This analysis was based on internal scrutiny and management of Telethon grants, publications acknowledging the Telethon grants, and information nationally or internationally publicly available. Despite an accurate monitoring of the results of each project after grant completion to understand its impact on patients and healthcare, the information reported may be partial and the elaborated picture incomplete.

For privacy reasons, the authors did not have direct access to the individual Customer Satisfaction feedbacks of patients admitted at the NEMO clinical centres. Information in the form of aggregated data was publicly available from the NEMO annual Impact Report 2019 [9]; the authors are not responsible for the methods applied by professionals of the NEMO centres for the analysis of data derived from such Customer Satisfaction questionnaires.

## Conclusions

The funds awarded within the Telethon-UILDM calls and through topic-related ad hoc grants have contributed to support a network of physicians who actively collaborated on several clinical research projects, sharing their experience, and adopting common medical care and research approach to NMDs. The initial idea of Telethon and UILDM that led to the development of this program in 2001 proved to be very valid and visionary, as demonstrated by the consequent expansion and cultural growth of the Italian NMD clinical network thanks to this support. While training and development of scientific competences are matter for scientific societies, this strategic program has contributed to a paradigm shift on issues such as data sharing and on the implementation of patient-centric research and multidisciplinary care, at least in Italy. Furthermore, the increased clinical knowledge on NMDs contributed to stir international discussion within the disease community and with regulators, facilitating the development of innovative therapies. This demonstrates that charities and POs may synergise with other stakeholders and contribute to orphan medicine development even when these don’t derive from their own internal pipeline. Although each country has its own health research working models, sharing this experience and lessons learned can therefore be beneficial to other charities and POs that want to promote clinical research towards the care of patients with rare diseases.

The advent of innovative therapeutics has raised lot of hope, with therapies already available to patients for a few diseases, such as DMD and SMA. However, much work remains to be done. First of all, much information is still lacking for correct interpretation of trial results; issues like natural history expanded to a wider-age group, the role of confounding factors (such as genetic modifiers, multi-organ involvement, etc.), and biomarker validation require further investigation. Furthermore, one must consider that NMDs are part of a broad category of diseases; clinical characterisation and natural history are still lacking for many of them, and the experience gained on DMD and SMA should guide research on other NMDs as well. Finally, we must be sought for the daily medical needs of those patients who do not fully benefit from advanced therapies, as they are treated when the disease is no longer at a very early stage or do not have access to these therapies, due to their genotype or for other reasons. While these topics may require even more strict definition of priorities within the calls for research proposals, the investigator-driven nature of this funding scheme remains very valid and can help attract innovative (and sometimes unpredictable) ideas and new expertise into the field.

## Data Availability

The PubMed Identifier dataset used for the analyses reported in the text and in Tables 2, 3 and 4 is available from the corresponding author. Please contact the corresponding author for data requests.

## Abbreviations

CMT: Charcot–Marie–Tooth disease
cTAP: Collaborative Trajectory Analysis Project
DMD: Duchenne muscular dystrophy
NEMO: NEuroMuscular Omni centre
NIH: National Institutes of Health
NMD: Neuromuscular disorder
OM: Outcome measure
PO: Patient organisation
QoL: Quality of life
RCR: Relative citation ratio
SMA: Spinal muscular atrophy
TREAT-NMD: Translational Research in Europe—Assessment and Treatment of NeuroMuscular Diseases
TTR: Transthyretin
UILDM: Unione italiana lotta alla distrofia muscolare
